# A Retrospective Study on Clinical Features and Visual Outcome of Patients Hospitalized for Ocular Trauma in Cangzhou, China

**DOI:** 10.1155/2017/7694913

**Published:** 2017-03-12

**Authors:** Xi Zhang, Yuqing Liu, Xiangning Ji, Yuanyuan Zou

**Affiliations:** Department of Ophthalmology, Cangzhou Central Hospital, No. 16, Xinhua Road, Yunhe District, Cangzhou, Hebei 061000, China

## Abstract

*Purpose*. To describe clinical features and to analyze visual outcome of ocular trauma in Cangzhou in 2012–2015, China. *Methods*. A retrospective study of ocular trauma cases admitted to Cangzhou Central Hospital from January 2012 till December 2015 was performed. *Results*. This study included a total of 507 eyes from 478 patients. Four hundred (83.7%) patients were male, with a male-to-female ratio of 5.1 : 1. Mean age was 43.6 ± 18.3 years (5–95 years). The largest age group was 45–59 years old, followed by 30–44 years old, presenting two peaks of the age distribution and accounting for 28.5% and 27.2%, respectively. The most frequent type of injuries was work-related (194, 40.6%) followed by home-related (123, 25.7%). Initial visual acuity (VA) correlated with final VA (Spearman's test, *r* = 0.703, *p* = 0.001). The Ocular Trauma Score also correlated with the final VA significantly (Spearman's test, *r* = 0.802, *p* = 0.001). *Conclusions*. Susceptible population of eye injuries were middle- and young-aged working groups, and the proportion of males was higher. The leading two types of ocular trauma were work-related and home-related. Initial VA was a significant predictor of the final VA and the OTS possibly had predictive value in the final VA.

## 1. Introduction

Ocular trauma is one of the main causes of severe ocular morbidity [1–8]. Globally, more than 55 million eye injuries occur per year, while there are approximately 1.6 million people with blindness from ocular trauma, 2.3 million people who are bilaterally visually impaired, and 19 million people with unilateral blindness or visual loss [9].

Decrease or loss of vision, either monocular or binocular, may result in significant economic burdens to families and countries due to time lost from work, or school, and family care giving, expensive hospitalization, special visit and treatment, prolonged follow-up, and visual rehabilitation. Because of the severity of visual impairment of ocular trauma, complete ocular trauma statistics and authoritative data should be collected.

The epidemiology of ocular trauma has been well described in developed countries such as the United States, the UK, Australia, Japan, and Europe [8, 10–13], which is very useful in defining the impact of ocular trauma. The incidence of ocular trauma may be higher in developing countries.

The goals of this study were to describe clinical features of ocular trauma, to analyze visual outcomes of ocular trauma, and finally to make recommendations for public health and clinical strategies for the prevention, management, and research of ocular trauma in the future.

## 2. Materials and Methods

Patients with ocular trauma admitted to Cangzhou Central Hospital (CCH) from January 2012 till December 2015 were reviewed retrospectively. CCH is one of the biggest general medical center, serving more than 7 million people in urban and suburban areas of Cangzhou. Excluded from the analysis were 13 patients with previous ocular surgeries or pre-existing ocular conditions affecting VA and 34 cases with less than 6 months of follow-up.

Age, gender, the affected eye, causes and types of ocular trauma, time interval from injury to presentation, duration of hospitalization, and follow-up were recorded. Initial visual acuity (VA), management, and final VA were further documented. Ages were divided into five groups as 0–14, 15–29, 30–44, 45–59, and ≥60 years. Ocular trauma was classified by the standardized international classification of ocular trauma, Birmingham Eye Trauma Terminology system (BETTS) [14–16]. Types of ocular trauma were divided into six types as work-related, home-related, school-related, sports-related, traffic-related, and violence-related. Time interval from injury to presentation was divided into four groups as 0–24 hours, 24–48 hours, 2–4 days, and ≥4 days. Initial and final VAs were both classified under no light perception (NLP), light perception (LP)/hand motion (HM), 1/200–19/200, 20/200–20/50, and ≥20/40.

We also used the Ocular Trauma Score (OTS) [16] to evaluate the severity of ocular trauma and to analyze the correlation between OTS and final VAs. This index allows prediction of the visual outcome in patients with ocular trauma according to certain numerical values (initial VA, rupture, endophthalmitis, perforating injury, retinal detachment, and afferent pupillary defect) at presentation. The likelihood of the final VAs in the OTS categories (one through five) in this study was calculated and compared with those in the OTS study.

Statistical analysis was performed using SPSS version 16.0 statistical software (IBM, Armonk, NY, USA). Categorical and continuous data were expressed in the form of proportion and mean ± SD (standard deviation), respectively. Pearson's chi-squared test and Student's *t*-test were used to evaluate differences in parametric variables. Correlation analyses (between initial VA and final VA and between OTS and final VA) were performed using Spearman's test. A *p* value less than 0.05 is considered statistically significant.

## 3. Results

This 4-year study of ocular trauma seen in CCH included 507 injured eyes in 478 patients (154 cases in 2012, 109 cases in 2013, 103 cases in 2014, and 112 cases in 2015), with no significant change in annual rates of ocular trauma during the four-year period. Males had a higher rate than females (83.7% versus 16.3%), with a male-to-female ratio of 5.1 : 1. Mean age was 43.6 ± 18.3 years (5–95 years). The largest age group was 45–59 years followed by 30–44 years, presenting two peaks of the age distribution and accounting for 28.5% and 27.2%, respectively. Two hundred and thirty (48.1%) patients had injuries in their left eyes whereas 219 (45.8%) had injuries in their right eyes. Both eyes were involved in 29 (6.1%) patients. Of the patients, 80.5% presented within 24 hours after eye injuries. A further 5.9% presented between one and four days from the occurrence of the injury. Only 13.6% presented more than 4 days after sustaining the eye injuries. Average duration of hospitalization was 14.7 ± 11.0 days (0–68 days). Mean duration of follow-up was 10.4 ± 2.3 months (6.1–12.1 months). Open globe injury, accounting for 62.5%, was the most common eye injury, which included 34 cases of rupture, 197 case of penetrating injury, 83 cases of intraocular foreign body, and 3 cases of perforating injury. Closed globe injury was the second one with 32.1%, which included 151 cases of contusions and 12 cases of lamellar lacerations. Two hundred and ninety-five (52.8%) eyes had adnexa injuries (Table 1).

**Figure 1:**
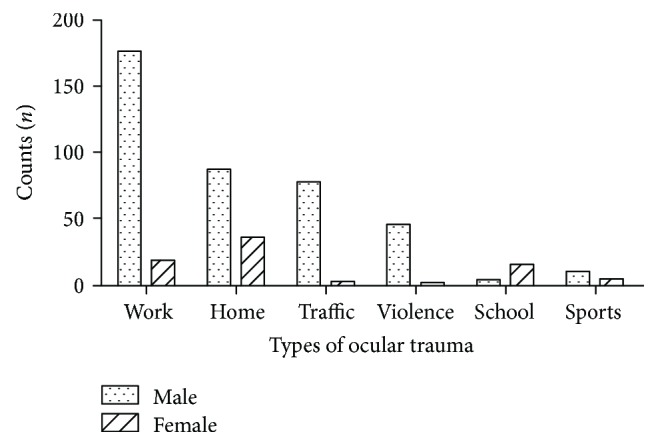
Types of ocular trauma.

Work-related injuries accounted for the majority of ocular trauma (194, 40.6%), followed by home-related (123, 25.7%), traffic-related (89, 18.6%), violence-related (41, 8.6%), school-related (17, 3.6%), and sports-related (14, 2.9%). In males, the most frequent type of ocular trauma was work-related (176, 44.0%) followed by home-related (87, 21.8%) and traffic-related (78, 19.5%). In contrast, in females, home-related injuries accounted for the largest proportion (36, 46.2%) followed by work-related injuries (18, 23.1%) and school-related (15, 19.2%). Sports-related injuries accounted for 2.5% in males and 5.1% in females. Types of ocular trauma differed significantly in gender (Pearson's chi-squared test, *p* < 0.001) (Figure 1).

There was a wide variety of injury causes that resulted in ocular trauma. Most of the injuries were caused by metallic objects (119, 24.9%). Metallic objects were the main cause in all age groups except for patients aged 0 to 14 years old, for whom the main causes were firework and sharp objects. Traffic accidents were seen mostly in patients aged between 15 to 29 years old, and blunt objects injuries were seen the most commonly in aged 30 to 44 years group. Injuries caused by falling was seen most commonly for the elderly. Causes of injuries differed significantly in different age groups (Pearson's chi-squared test, *p* < 0.001) (Table 2).

In terms of management, 53 (10.5%) eyes were medically treated, and the rest 454 (89.5%) eyes required surgical intervention. Ocular wall repair (286, 56.4%), lensectomy, or phacoemulsification (172, 33.9%) were the most common surgical procedures. Posterior vitrectomy was required for 120 (23.7%) eyes with posterior foreign bodies, vitreous hemorrhages, retinal detachments, and endophthalmitis. Removal of foreign body was performed in 83 (16.4%) eyes. Anterior chamber washout was needed for 45 (8.9%) eyes due to severe hyphema and anterior chamber foreign bodies. Thirteen (2.6%) enucleations were performed, due to uncontrolled endophthalmitis and severe open globe injuries, including five primary and eight secondary enucleations (Table 3).

Endophthalmitis occurred in 3 eyes (0.6%). All of the injured eyes had the infection's signs at the time of initial presentation. The strongest association with these was the mechanism of injury, which included an accidental lesion with a piece of wood, a punch, and a piece of a bird's feature. The three cases all had vitrectomy and received intravitreal antibiotics at the primary repair. Although treated aggressively, final VA was still poor, which achieved HM or LP after about 6 months of follow-up.

**Figure 2:**
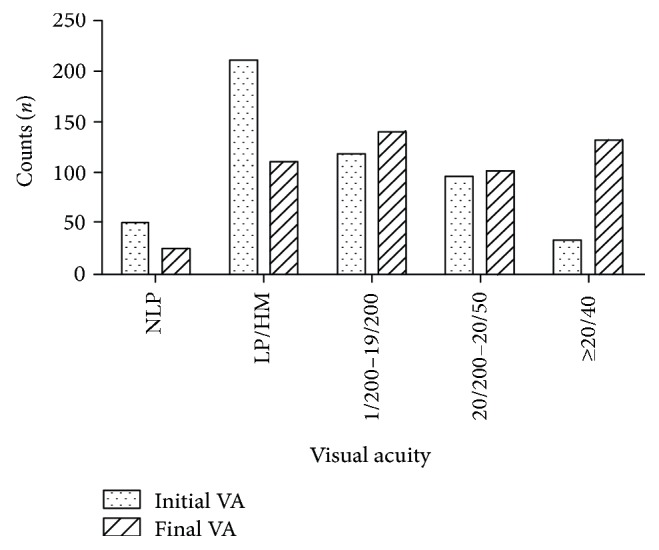
Visual acuity.

A comparison between initial VA and final VA is illustrated in Figure 2. Only thirty-three (6.5%) eyes had initial VA of 20/40 or better, 96 (18.9%) eyes had initial VA of 20/200–20/50, 118 (23.3%) eyes had initial VA of 1/200–19/200, 210 (41.4%) eyes had initial VA of LP/HM, and the rest 50 (9.9%) eyes had initial VA of NLP. After about 6 months of follow-up, the final VA was 20/40 or better in 132 (26.1%) eyes, 20/200–20/50 in 101 (19.9%) eyes, and 1/200–19/200 in 140 (27.6%) eyes. One hundred and ten eyes (21.7%) had final VA of LP/HM and 24 (4.7%) eyes had final VA of NLP. There was a significant correlation between initial VA and final VA (Spearman's test, *r* = 0.703, *p* = 0.001).

The percentage of the final VA by the OTS score in comparison with the OTS study is shown in Table 4. Five hundred and seven eyes were classified within OTS categories one through five. Against USEIR-OTS system, our study had a smaller sample size; we still could see a close resemblance between the scores in our study and that in the USEIR study of OTS. The OTS correlated with final VA significantly (Spearman's test, *r* = 0.802, *p* = 0.001).

## 4. Discussion

In our study, males had a higher rate than females, especially those under 65 years old. Other studies also reported a higher rate in males [17–21]. This might be due to different occupational exposure between different genders. In China, most females are housewives and engaged in occupations with low risk; however, males are prone to do rough work and more likely to take part in dangerous sports and activities. Mean age was 43.6 ± 18.3 years (5–95 years). Most of the eye injuries were found in middle-aged groups (45–59 years) followed by young-aged groups (30–44 years) which is coincident with other studies [2, 22]. This may be explained by the fact that the working population is of high risk and accounts for the largest portion of ocular trauma. In China, people between the age of 30 to 44 years old and 45 to 59 years old are exactly the major labors and play major roles in supporting families, resulting in a significantly larger portion than others.

After injuries, over four-fifths of patients (385, 80.5%) presented on the same day as sustaining their injuries, and less than one-seventh of patients (65, 13.6%) still had a delay of 4 days before clinical review. Cao et al. [4] thought that delayed presentation was a matter of concern about final VAs. This suggests that the public's awareness of seeking medical care in timely manners should be improved.

In our study, average duration of hospitalization was 14.7 ± 11.0 days that was longer than most studies which had a mean of 8.4 days [4]. This could be related to the severity of injuries or social reasons. Firstly, due to local health care system in our region, only relatively serious eye injuries require hospitalization, which needed multiple operations within longer hospital admission. At the same time, rural patients accounted for the majority of the injured patients, and they preferred to stay in hospital before obtaining stable vision under the covering of the system of industrial injury insurance, which resulted in the extension of hospital admission. Secondly, the lengthy admissions of more than 40 days partly occurred in patients who had sustained severe chemical burns. These patients required multidisciplinary care with plastic surgery involvement for skin grafting. Burns was a rare cause of ocular morbidity, but it resulted in the longest hospital admission.

In our study, work-related injuries (40.6%) were the most common type of ocular trauma, and metallic objects, such as wire, steel, hammering, or grinding wheel, widely used in workplaces, remained the leading (24.9%) agents that cause eye injuries, which were consistent with other studies [4, 23]. The main contributing factor for the higher proportion of work-related injury is the local work tasks including grinding, welding, hammering, drilling, metal cutting, and nailing, which commonly involve high-powered tools that generate metal fragments at high velocities. When the objects shoot people, the effective action area is small. But with the hard body, the energy it delivered is very large and often has devastating effects on the eyes. However, another important factor is disregarding the safety of workers. In addition, we also found that home-related injuries were the second predominant (25.7%) eye injury. In our region, most females and children tend to spend more time at home and may be more likely to do some “do-it-yourself” tasks or be cleaning the house with the use of tools and machinery for home improvement, resulting in damage on the eyes. Overall, the leading two types of ocular trauma in our study were work-related and home-related, which corresponds to results described in previous studies [24, 25]. These data suggest a need to establish, implement, and monitor compliance to guidelines in occupational safety and health, and also highlight a need to educate the public about safety precaution use of protective eye wear not only at workplaces, but also in the home.

With regard to surgical procedures, of the 507 injured eyes, 56.4% had ocular wall repairs, 33.9% had lensectomy or phacoemulsification, and 8.9% had anterior chamber washouts, which suggest that injury is preferred in the anterior segment of the globes than the posterior segment, highlighting the significance of the anterior segment. This not only suggests that wearing eye-protection devices should be introduced, because such anterior segment injuries would have been easily blocked by eye-protection devices [26], but also suggests that the training modules for the emergency and junior ophthalmologists should emphasize the anterior segment of the eye globe, types of ocular trauma, appropriate precautions, and first-aid management of the injured eyes.

In the current study, of the 50 eyes with initial VA of NLP, 26 eyes ended up with improved vision including 2 eyes which obtained final VA of better than 20/200, 24 eyes achieved final VA of less than 20/200, and the rest 26 eyes still had VA of NLP at the last follow-up. Of the 129 eyes with initial VA of 20/200 or better, 87 (67.4%) eyes had final VA of 20/40 or better. Among the 378 eyes with initial VA of less than 20/200, only 45 (11.9%) eyes had final VA of 20/40 or better. Those eyes with final VA of NLP all occurred in cases with initial VA of LP/HM or worse. Meng and Yan [27] have demonstrated that initial VA was found to correlate significantly with final VA in eye injuries. Our study showed the same result that initial VA correlated with final VA (Spearman's test, *r* = 0.703, *p* = 0.001), which was consistent with that of Man and Steel [28] and Weichel et al. [29].

As for OTS score, except for OTS-two where final VA of 20/200 or better was predicted in 8% of patients in our study as against 28% in OTS study and OTS-three where final VA of 1/200–19/200 or worse was in 9% of patients in our study against 28% in OTS study, our results were similar to those in the OTS study signifying the clinical importance and practical application of OTS. Kuhn et al. [16] have demonstrated that the patient with OTS score of one will have a higher risk of poorer final VA as against the patient with OTS score of five who will have higher probability of better final VA. Our study shows the similar results that only 6% of eyes with OTS-one had a final VA of 20/200 or better whereas 47% had a final VA of NLP; however, the eyes with OTS-five 97% had a final VA of 20/40 or better. Higher OTS scores tend to indicate a better prognosis. OTS correlated with final VA significantly (Spearman's test, *r* = 0.802, *p* = 0.001). The OTS had high prognostic accuracy and could be used in counselling patients and in management decision making after injury.

There were several limitations in our study. First, the number of injured eyes might be underestimated. This may be because of some closed globe injuries, which might be treated as outpatients. Second, comprehensive information about medical records could not be acquired, such as the extent of injury, zone involvement intraoperatively, and the time to first surgery in cases of open globe injuries, so we were unable to reorganize the correction between the above factors and final VA. This suggests that, a standardized reporting system, United States Eye Injury Registry (USEIR) surveillance, as exists in other countries [30], is recommended and would help to evaluate changes in the epidemiology of eye injuries over time and provide population-based longitudinal data for preventive strategies. However, with these limitations, our data still provided useful information concerning the clinical characteristics of ocular trauma.

In conclusion, susceptible population of eye injuries were middle- and young-aged working groups, and the proportion of males was higher. The leading two types of ocular trauma were work-related and home-related. Initial VA was a significant predictor of the final VA and the OTS possibly had a predictive value in final VAs in ocular trauma. It is therefore recommended that efforts should be invested in education for eye protection in order to prevent ocular trauma in the young- and middle-aged working groups, and that eye injuries research and prevention could be further aided by a nationwide collaborative registry of eye injuries in China.

## Figures and Tables

**Table 1:** Characteristics of patients with ocular trauma.

Variables	*n*
Total patients/total injured eyes	478/507
Left/right/both eyes	230/219/29
Male/female	400/78
Age (years, mean ± SD)	
Total	43.6 ± 18.3
Male/female	41.8 ± 16.8/51.5 ± 22.9
0–14	19
15–29	105
30–44	130
45–59	136
≥60	88
Mean duration of follow-up (months)	10.4 ± 2.3
Mean duration of hospitalization (days)	14.7 ± 11.0
Time interval from injury to presentation	
0–24 hours	385
24–48 hours	16
2–4 days	12
≥4 days	65
Diagnosis	
Open globe injuries	317
Penetrating	197
IOFB	83
Perforating	3
Rupture	34
Closed globe injuries	163
Contusion	151
Lamellar laceration	12
Chemical burn confined to the eye and adnexa	14
Thermal burn confined to the eye and adnexa	13
Adnexa injuries of globe	295

SD: standard deviation; IOFB: intraocular foreign body.

**Table 2:** Distribution between causes of injuries and age groups.

Causes of injuries	Age groups (years)					
	0–14	15–29	30–44	45–59	≥60	Total
Metallic objects	1	29	33	45	11	119 (24.9%)
Wood	2	5	8	10	3	28 (5.9%)
Blunt objects	3	10	21	13	11	58 (12.1%)
Traffic accidents	0	31	25	23	10	89 (18.6%)
Chemical	0	1	5	5	1	12 (2.5%)
Firework	5	6	9	10	4	34 (7.1%)
Finger/fist	1	10	7	12	1	31 (6.5%)
Falling	1	2	10	5	35	53 (11.1%)
Sharp objects	6	11	12	13	12	42 (8.8%)
Total	19 (4.00%)	105 (22.0%)	130 (27.2%)	136 (28.4%)	88 (18.4%)	478 (100%)

**Table 3:** Management of the 507 injured eyes from 478 patients.

Management	*n*	%
Nonsurgical	53	10.5
Surgical	454	89.5
Ocular wall repair	286	56.4
Lensectomy or phacoemulsification	172	33.9
Posterior vitrectomy	120	23.7
Removal of foreign body	83	16.4
Anterior chamber washout	45	8.9
Orbital fracture repair	30	5.9
Canalicular anastomosis	32	6.3
Glaucoma surgery	15	3.0
Enucleation	13	2.6
Keratoplasty or amniotic membrane transplantation	9	1.8

**Table 4:** Percentage of the final VA by the OTS score in comparison with the OTS study.

Raw OTS	OTS	NLP	LP/HM	1/200–19/200	20/200–20/50	≥20/40
0–44	1	47/73	37/17	10/7	6/2	0/1
45–65	2	12/28	50/26	30/18	8/13	0/15
66–80	3	1/2	3/11	5/15	47/28	44/44
81–91	4	0/1	2/2	1/2	37/21	60/74
92–100	5	0/0	0/1	1/2	2/5	97/92
